# Identification of transcriptional biomarkers by RNA-sequencing for improved detection of β_2_-agonists abuse in goat skeletal muscle

**DOI:** 10.1371/journal.pone.0181695

**Published:** 2017-07-26

**Authors:** Luyao Zhao, Shuming Yang, Yongyou Cheng, Can Hou, Xinyong You, Jie Zhao, Ying Zhang, Wenjing He

**Affiliations:** Key Laboratory of Livestock-product Quality and Safety Research Division, Institute of Quality Standards and Testing Technology for Agro-Products, Chinese Academy of Agricultural Sciences (CAAS), Beijing, PR China; Huazhong University of Science and Technology, CHINA

## Abstract

In this paper, high-throughput RNA-sequencing (RNA-seq) was used to search for transcriptional biomarkers for β_2_-agonists. In combination with drug mechanisms, a smaller group of genes with higher detection accuracy was screened out. Unknown samples were first predicted by this group of genes, and liquid chromatograph tandem mass spectrometer (LC-MS/MS) was applied to positive samples to validate the biomarkers. The results of principal component analysis (PCA), hierarchical cluster analysis (HCA) and discriminant analysis (DA) indicated that the eight genes screened by high-throughput RNA-seq were able to distinguish samples in the experimental group and control group. Compared with the nine genes selected from an earlier literature, 17 genes including these nine genes were proven to have a more satisfactory effect, which validated the accuracy of gene selection by RNA-seq. Then, six key genes were selected from the 17 genes according to the variable importance in projection (VIP) value of greater than 1. The test results using the six genes and 17 genes were similar, revealing that the six genes were critical genes. By using the six genes, three positive samples possibly treated with drugs were screened out from 25 unknown samples through DA and partial least squares discriminant analysis (PLS-DA). Then, the three samples were verified by a standard method, and mapenterol was detected in a sample. Therefore, the six genes can be used as biomarkers to detect β_2_-agonists. Compared with the previous study, accurate detection of β_2_-agonists abuse using six key genes is an improvement method, which show great significance in the monitoring of β_2_-agonists abuse in animal husbandry.

## 1. Introduction

Abuse of β_2_-agonists in animal husbandry has become a common practice in recent years. Among mainstream methods of detecting drug residues [1,2], instrumental analysis, immunoassays, sensor- and chip-based analysis have received tremendous attention, and substantial improvement has been made on them. These methods, however, are all based on known β_2_-agonist drugs. New drugs and unregulated drugs are overlooked by regulatory departments, which continuously impose threats on the safety of consumers. Therefore, it is necessary to develop a new screening technique for effective large-scale monitoring of homogeneous drugs, especially new drugs.

With rapid development of various -omics technologies, information can be extracted from -omics data using statistical tools on the same platform, making it possible to find out highly effective and sensitive biomarkers. Previous studies have shown that β_2_-agonists affect gene expression in different tissues and organs, providing a basis for biomarkers search at the transcriptional level. In organisms, β_2_-agonists combined with β_2_-adrenergic receptors trigger the coupling between receptors and guanine nucleotide-binding protein (G protein). In this way, adenylate cyclase (AC) can be activated, which converts adenosine triphosphate (ATP) into 3,5-cyclic adenosine monophosphate (cAMP) and causes a series of in vivo reactions [3–5]. β_2_-agonists also help regulate the concentrations of Na^+^ and Ca^++^ in cells [6,7], treat inflammation [8,9] and relax smooth muscle [10]. Ractopamine reportedly induced the change in the expression amount of β_2_-adrenergic receptor (β_2_-AR) in porcine smooth muscle [11], as well as the expression level of fatty acid synthase (FAS) in adipose tissue of fattening pigs [12]. Zilpaterol may trigger the variation in the expression of β_2_-AR in cattle muscle tissues [13]. The use of clenbuterol alter the expression of apolipoprotein R (apoR) in porcine fat cells and bovine smooth muscle cells [14], in addition to the abundances in the insulin-like growth factor-1 (IGF-1) in gracilis cells of mice [15], β_2_-AR in muscle cells of the left ventricles of mice [16], SOCS box protein 15 (ASB15) in longissimus muscle on the cattle back [17] and muscle growth inhibitor (mRNA) in breast muscle cells of chickens [18]. After ractopamine was fed to pigs, IGF-1 expression in smooth muscle cells was changed [19]. By using different β_2_-agonists to treat animal subjects, the preceding researches aim to verify the genes that cause obvious differences in the expression. However, using these genes as biomarkers to monitor β_2_-agonists abuse has not yet been reported.

The aim of this study is to find biomarkers for β_2_-agonists at the transcription level, which are expected to monitor misuse of β_2_-agonists in livestock husbandry to compensate for regulatory flaws. In the earlier research [20], nine genes selected from literatures including β_2_-adrenergic receptor (ADRB2), cAMP-dependent protein kinase (PRKACB), adenylate cyclase 3 (ADCY3), Na^+^/K^+^-ATPase (ATP1A3), Ca^++^-ATPase (ATP2A3), parathyroid hormone (PTH), myosin light chain kinase (MYLK), tumor necrosis factor-α (TNF-α) and interleukin-1β (IL1B) were used to differentiate experimental group samples from control group samples. The relative expression fold and changing trend of each gene were consistent with the mechanism of β_2_-agonist drugs. The correlation analysis results showed that the predicted values using the regression formula established based on the nine genes and actual sample residual values were highly correlated with the actual residual values of the samples. The results indicated that only ractopamine caused changes in the expressions of the nine genes, which could be used as biomarkers to indicate the use of ractopamine. Because only ractopamine was used in animal experiments to treat goats, further investigation is required to determine whether this group of genes is effective in monitoring other β_2_-agonist drugs.

In this study, RNA-sequencing was used as a non-targeted-omics technique to optimize the group of genes selected through a targeted -omics method [20]. Compared with qRT-PCR, RNA-seq has higher detection throughput and a wider detection range, which provides a possibility of finding more biomarkers. To find more accurate biomarkers at the transcriptional level for β_2_-agonist drug misuse monitoring, this study improved the previous study [20] with a high-throughput RNA-seq technology. This approach is expected to further screen target genes to obtain more stable, sensitive and efficient biomarkers for higher detection precision. Biostatistical methods like principal component analysis (PCA), hierarchical clustering analysis(HCA) and discriminant analysis(DA) were applied to test if the selected genes could get a clear distinction between 27 treatment and 27 control samples. This study also used VIP (Variable Influence on the Projection) values to screen potential biomarkers to determine key factors among the selected genes, and genes with VIP>1 are considered to have critical influence on the model according SIMCA-p, which were expected to reduce the monitoring cost and simplify operations. The six key factors were then employed to predict whether drugs were used in 25 unknown samples through DA and partial least squares discriminant analysis(PLS-DA). Subsequently, a standard method (*Detection of β-receptor agonist residues in animal-source food by LC-MS/MS*, *Notice #1025-18-2008*, *Ministry of Agriculture*, *China*) was used to verify the positive samples and determine the drug types and names, which would prove the effectiveness of the potential biomarkers.

## 2. Materials and methods

### 2.1. Ethics statement

Animals researches in this study were conducted in accordance with the guidelines for animal experiments of the Feed Research Institute, Chinese Academy of Agricultural Sciences, Beijing, China. Animal care activities were approved by the International Cooperation Committee of Animal Welfare (ICCAW), Beijing, China.

### 2.2. Animal experiments and sample collection

Fifty-four healthy male goats weighing 30±2.5 kg were selected and evenly divided into control and experimental groups. Before the experiment, blank feedstuff was given to the goats for one-week observation. Ractopamine was orally given to goats every morning at 1 mg/kg (body weight) in the experimental group before feeding for 28 consecutive days (dosing days). Then, the additive was canceled for 21 consecutive days (withdrawal days) until the end of the experiment. Three animals were sacrificed under local anaesthesia separately on doing days 7, 14 and 21 and on withdrawal days 0, 1, 3, 7, 14 and 21 after drugs were no longer fed (days). About 5g meat cut from the longissimus muscle on the back, washed with sterile phosphate buffer to remove fat tissues and connective tissues, and immediately moved to liquid nitrogen. Then, they were stored under –80°C until analysis. To verify the effectiveness of the selected target genes, muscle tissue samples of 25 healthy male goats were randomly obtained from slaughter lines in Dongsheng Slaughter House (Bayannaoer, Inner Mongolia, China). After the animals were slaughtered, 100 mg of longissimus muscle tissue on their backs was immediately placed into RNAlater (Tiangen Biotech, Beijing, China) and then stored under –80°C until analysis.

### 2.3. Gene expression level analysis

TRIzol^®^ reagents (Invitrogen, CA, USA) were used for RNA extraction following specified instructions. The total RNA concentration was measured by NanoDrop 2000c UV spectrophotometer (Thermo Scientific, Massachusetts, USA) and the OD260/280 value was recorded to determine the RNA purity. Agilent 2100 Bioanalyzer (Agilent, CA, US) was used to assess RNA integrity, with the RNA integrity number (RIN) used as an RNA quality parameter.

The first chain of cDNA was synthesized using SuperScript VILO cDNA synthetic reagent kit (Invitrogen, CA, US) according to the manufacturer's instructions to acquire the final total RNA concentration. In this manner, the total RNA addition volume was determined as about 1 μg. In the reaction system, 4 μL of 5X VILO^™^ reaction mix and 2 μL of 10X SuperScript^®^ enzyme mix were added before DEPC-treated to obtain 20 μL solution. The reaction was performed under 25°C for 10 min, 42°C for 60 min, 85°C for 5 min, then cDNA was stored at -20°C until use.

To select target genes, on the 21^st^ dosing day and third and 21^st^ withdrawal days, high-throughput RNA-seq was performed on the experimental group samples and corresponding control group samples with Illumina HiSeq^™^ 2500. The criteria for selecting target genes were: high statistical significance (p < 0.01); relative expression fold (x-fold, experimental group/control group) was lower than 0.6 or higher than 3 when compared with the control group; x-fold values were not significantly different on the 21st feeding day, 3rd withdrawal day, and 21st withdrawal day, which guaranteed long-term validity and stability of target genes. In addition, the gene signal pathways were considered to ensure the specificity of genes to the largest extent [21–23].

According to the nucleic acid sequence numbers of goats recorded on GeneBank (http://www.ncbi.nlm.nih.gov/nuccore), primers were designed using Primer Premier 5.00 (Premier, Ottawa, Canada) and Primer3 (http://bioinfo.ut.ee/primer3-0.4.0/) and synthesized by Sangon Biotech (Shanghai) Co., Ltd, as listed in Table 1.

**Table 1:** Sequencing-based selection of gene primer sequences and annealing temperatures.

Gene group	Gene	Primer sequence (5' to 3')	Annealing temperature	Product length
**enzyme**	***PDE4C***	**For** **AACGGGACCTGCTCAAGAC**	**60°C**	**112 bp**
		**Rev** **GCGTGTAGGCTGTTGTGGTA**		
	***HSL***	**For** **AGAGGGTCATTGCCGACTT**	**60°C**	**160 bp**
		**Rev** **TCGTTGCGTTTGTAGTGCTC**		
**Structural protein**	***FABP5***	**For** **GAGAAGTTTGAAGAGACCACAGC**	**60°C**	**105 bp**
		**Rev** **GCTTTCCTTTCCATCCCATT**		
	***LOC102185492***	**For** **CTCCAAACAGAGCAACAGCA**	**60°C**	**134 bp**
		**Rev** **AGGGCTTCACTGTCTTCGTC**		
	***MYH9***	**For** **GCCTGTTCTGTGTGGTCATC**	**59°C**	**127 bp**
		**Rev** **GCGGTGTCTGTGATAGCGTA**		
**Modulating protein**	***NEB***	**For** **CAAGAGGGCTTACTGGAACG**	**55°C**	**121 bp**
		**Rev** **GTCTGTGACTGTGCGATGGT**		
	***CREM***	**For** **ACCACAGCCATCCGTTATTC**	**59°C**	**121 bp**
		**Rev** **CCAGGCACATCAGAGGAAAG**		
	***FOXO1***	**For** **TCCAGCCAGAGCAGTATTTG**	**59°C**	**140 bp**
		**Rev** **GATTGAGCATCCACCAGGA**		
**Reference gene**	***GAPDH***	**For** **GCAAGTTCCACGGCACAG**	**60°C**	**195 bp**
		**Rev** **GGTTCACGCCCATCACAA**		

Real-time quantitative PCR 7500 system (Applied Biosystems, CA, US) was used to measure the relative expression amounts of candidate genes. SYBR^®^Premix Ex Taq^™^II (Tli RNaseH Plus; TAKARA, Dalian, China) was applied in the reaction according to the instructions of the reagent kit. The materials in the system were 10 μL of SYBR^®^ Premix Ex Taq II, 0.8 μL of primers (10 μM), 0.8 μL of reverse primers (10 μM), 6 μL of double-distilled water (ddH_2_O) and 2 μL of cDNA. The reaction was performed in an 8-strip tube (Axygen, Silicon Valley, USA) with a pipette gun epMotion5075 (Eppendorf).

The following real-time PCR cycling protocol was employed in two steps. First, pre-denaturation was performed at 95°C for 30s. Then, denaturation was performed at 95°C for 5s and reaction was allowed at the annealing temperature for 34s as described in Table 1 (40 cycles). The dissolving curve step was finally added on the PCR instrument. The threshold cycle (Ct) and melting curve were both obtained through the SDS software of real-time quantitative PCR 7500 instrument (Applied Biosystems, USA). Subsequent data analysis was performed with the melting curve characterized with only one peak, whereas data with irregular melting peaks were excluded from the quantification procedure. The relative quantitative method was used to process raw data using the following formulas:

△Ct=Ct(target gene)−Ct(reference gene)

△△Ct=△Ct(experimental group)−Average △Ct(control group)

### 2.4. Data processing

The expression ratio of the treatment group to the control group is expected as 2^-ΔΔCt^ and represents the x-fold regulation with a value of 1.00, indicating no expression change after the treatment. The measurement was conducted using the WPS software (Kingsoft, Beijing, China). SPSS 22.0 (IBM, CA, US) was used to perform data analysis aiming at determining genes with significant differences, which could be used as candidate biomarkers to identify drug dosing. In the Student-t test results, p ≤ 0.05 indicates a significant difference, and p ≤ 0.01 indicates a highly significant difference.

Biostatistical methods like PCA, HCA and DA were applied to test if the selected genes could get a clear distinction between 27 treatment and 27 control samples. PCA is a statistical procedure that uses an orthogonal transformation to convert a set of observations of possibly correlated variables into a set of values of linearly uncorrelated variables called principal components (PC). The normalized expression values of all responding genes were taken as the initial variables and reduced to two principal components only, thus facilitating resolution of treatment clusters in the scatter plot. By this, the data was reduced to a small number of dimensions (two dimensions here, PC1 and PC2) that can be plotted in a scatter plot. This transformation is defined in such a way that the first principal component (PC1) has the largest possible variance (that is, accounts for as much of the variability in the data as possible), and each succeeding component accounts for as much of the remaining variability as possible. Figures of PCA provide an intuitive way to show if there is a clear distinction between control and treatment groups. DA is a pattern recognition and machine learning method to find a combination of features that characterize or separate two or more classes of objects or events. Results of cross-validation demonstrate the effectiveness and reliability of the model.

PCA was carried out by SPSS 22.0 to verify the validity of the preceding candidate biomarkers. In addition, HCA and DA were used to verify the PCA results with different mathematical principles and presentation of results. Figures of HCA would display which sample go to the wrong tree. To screen key genes from the selected genes, SIMCA-P (Umetrics AB, Umea, Sweden) was used to obtain variable importance in projection (VIP) values to reduce the monitoring cost and simplify operations. According to the user guide of SIMCA-p, VIP provides the influence of every item in the matrix X on all the Y’s. Terms with VIP>1 have critical influence on the model.

After key genes were determined, DA was in coupled with PLS-DA by SIMCA-P to predict 25 unknown samples, which helped determine positive samples.

### 2.5. Result verification

The determined possibly drug-treated samples were further measured according to *Detection of β-receptor agonist residues in animal-source food by LC-MS/MS (Notice #1025-18-2008*, *Ministry of Agriculture*, *China)*. All reagents were of chromatographic grade and purchased from Fisher Scientific. 2 g (±0.01 g) of test samples were accurately weighed and placed into a 50 mL centrifugal tube, then 0.2 mol/L ammonium acetate solution (pH = 5.2) and β-glucuronidase/aryl sulfatase were added. After being evenly mixed, they were enzymolyzed in water bath in the dark. Extraction was carried out sequentially by perchloric acid (pH = 1±0.2) and ethyl acetate (pH = 9.5±0.2). After the upper-layer organic phase was removed, tert-butyl methyl ether was added to the lower-layer organic phase for extraction. The organic phases were combined and dried by nitrogen, then 2% formic acid solution was added. Subsequently, the solution passed through MCX solid-phase extraction column (60 mg/3 mL, Agilent, USA) and dried by nitrogen. And then it was dissolved in methanol-0.1% formic acid solution and measured by LC-MC/MC (UPLC/XevoTQ, Waters, US).

LC conditions: chromatographic column BEH C18 (50 x 2.1 mm, 1.7 μm, Agilent, New York, US); mobile phase A (0.1% formic acid-acetonitrile solution); mobile phase B (0.1% aqueous formic acid solution); gradient elution (0–2 min, 4% A; 2–6 min, 4–60% A; 12–12.1 min, 60–4% A; 12.1–16 min, 4–60% A); flow rate (0.3 mL/min); column temperature (30°C); sample volume (10 μL).

MS conditions: electrospray power supply; positive ion scan; multiple reaction monitoring (MRM); ionization voltage 3.2 kV; source temperature 110°C; atomizing temperature 350°C. All standards (> 98.0% purity) were purchased from Dr. Ehrenstofer GmbH Reagents Company (Germany). Monitoring ion pairs, collision energy, and declustering voltages were provided in S1 Table.

## 3. Results

### 3.1. Searching for accurate biomarkers by drug mechanisms coupled to RNA-sequencing

#### 3.1.1. RNA integrity

Agilent 2100 Bioanalyzer (Agilent, CA, US) was used to determine RNA integrity. The results showed that the RIN values of samples for RNA-seq were between 8.0 and 8.5, indicating that the RNA was complete and met the RNA-sequencing requirements; RIN values of the other samples were between 7.5 and 8.5, revealing that the RNA integrity met qRT-PCR requirements.

#### 3.1.2. RNA-sequencing

The total number of genes measured in the 21^st^ dosing day group is 686,092, and genes with significant expression differences from control is 39,256, accounting for 5.72%. The total number of genes measured in the third withdrawal day group is 632,279, and significantly different genes have a number of 11,275, accounting for 1.78%. The total number genes measured is 582,047 of the 21^st^ withdrawal day group, and genes with significant expression differences compared with control is 30,808, with a 5.29% proportion. The number of identical genes with significant expression differences from control are 11,269 for the first and second groups, 30,794 for the first and third groups, 11,181 for the second and third groups, and 3,808 for all the three groups. According to the selection criteria, the following genes were selected for qRT-PCR verification: phosphodiesterase 4C (PDE4C), hormone-sensitive lipase (HSL), fatty acid binding protein 5 (FABP5), immunoglobulin lambda peptide (LOC102185492), myosin heavy chain 9 (MYH9), nebulin (NEB), cAMP response element modulation (CREM), and forkhead box O1 (FOXO1).

#### 3.1.3. qRT-PCR for the screened results based on RNA-sequencing

qRT-PCR was performed on the 54 samples in the experimental and control groups to verify the eight genes screened by RNA-seq. The eight genes of the test group showed significant expression level differences from the control group, which validated the RNA-sequencing results. The qRT-PCR results indicated that two genes were obviously down-regulated while six genes were obviously up-regulated, which were consistent with the RNA-seq results. The p values and relative expression folds (x-fold) of all genes in the two groups verified by high-throughput RNA-seq and qRT-PCR were shown in Table 2.

**Table 2:** Comparison of RNA-seq results and qRT-PCR results (*x*-fold and *p* values of the selected eight genes).

Gene	X-fold value	X-fold value	*P* value	X-fold value	X-fold value	*P* value	X-fold value	X-fold value	*P* value
	RNA-seq	qRT-PCR	qRT-PCR	RNA-seq	qRT-PCR	qRT-PCR	RNA-seq	qRT-PCR	qRT-PCR
	Dosing-day-21	Dosing-day-21		Withdrawal-day-3	Withdrawal-day-3		Withdrawal-day-21	Withdrawal-day-21	
***PDE4C***	**9.872**	**1.328**	**0.015**	**6.129**	**1.244**	**0.001**	**6.158**	**1.188**	**0.022**
***HSL***	**0.138**	**0.194**	**0.007**	**0.471**	**0.642**	**0.037**	**0.306**	**0.778**	**0.041**
***FABP5***	**25.205**	**20.628**	**0.003**	**21.688**	**17.397**	**0.012**	**18.782**	**3.825**	**0.028**
***LOC102185492***	**10.736**	**6.666**	**0.025**	**10.834**	**7.005**	**0.006**	**7.805**	**1.604**	**0.019**
***MYH9***	**0.001**	**0.742**	**0.006**	**0.052**	**0.500**	**0.018**	**0.219**	**0.403**	**0.039**
***NEB***	**5.577**	**18.122**	**0.001**	**4.968**	**4.375**	**0.021**	**4.493**	**2.192**	**0.012**
***CREM***	**8.439**	**10.984**	**0.019**	**8.796**	**5.961**	**0.005**	**6.159**	**3.148**	**0.002**
***FOXO1***	**10.010**	**28.012**	**0.033**	**3.333**	**13.095**	**0.012**	**6.311**	**11.572**	**0.015**

**Fig 1:**
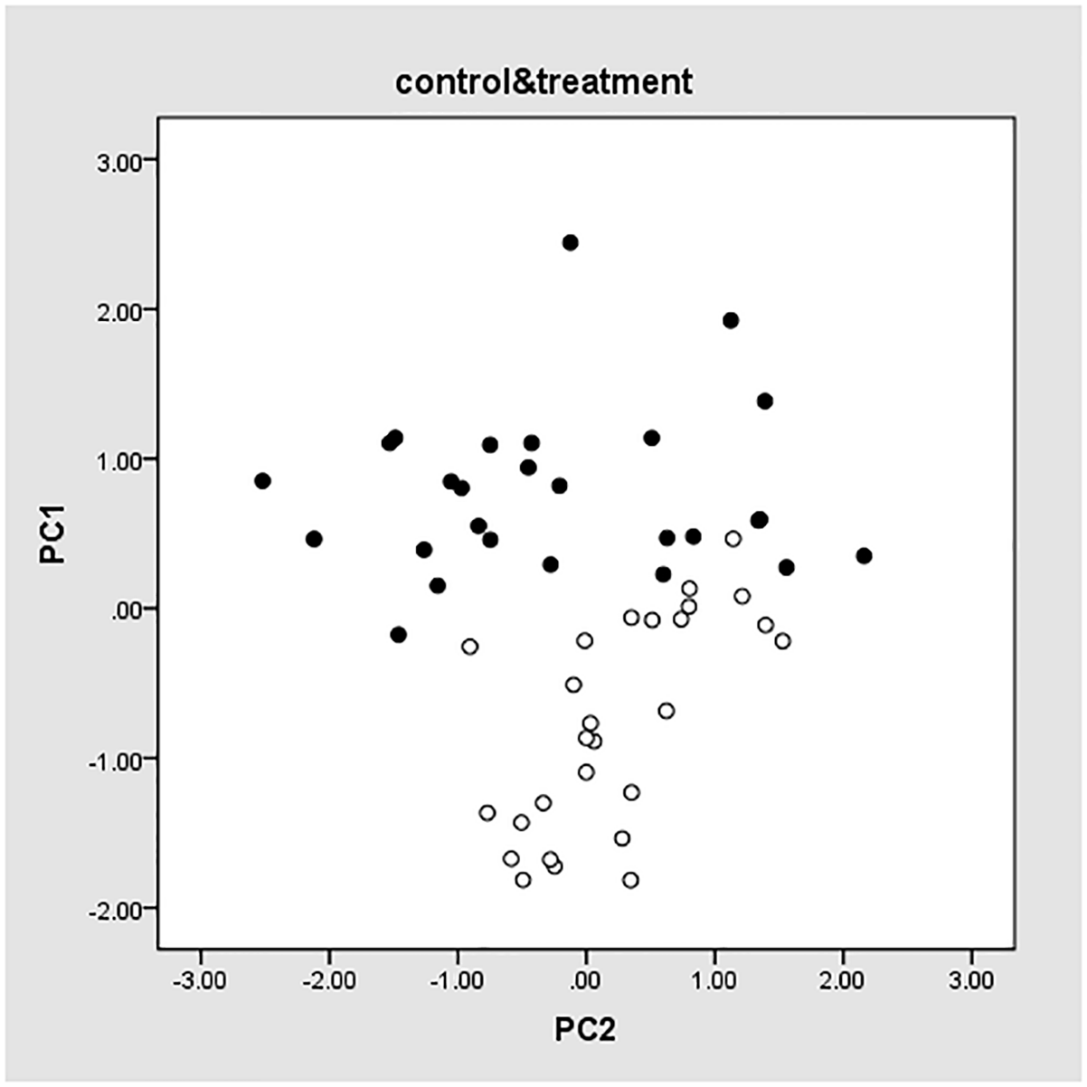
PCA values of eight genes.

To determine whether the selected eight genes showing can be used as potential biomarkers to differentiate treatment and control group, PCA was performed on the qRT-PCR results for all the samples selected on the 7^th^, 14^th^, and 21^st^ dosing days and 0, 1^st^, 3^rd^, 7^th^, 14^th^, and 21^st^ withdrawal days. The results were shown in Fig 1, where PC1 and PC2 were the obtained two principal components (PCs). Solid points represent samples in the control group, whereas hollow points represent samples in the test group. PC1 ranged from -1.88 to 0.46 for the test samples, and ranged from -0.23 to 2.48 for the control samples, indicating that the genes selected by RNA-seq were able to differentiate samples in the two groups.

**Fig 2:**
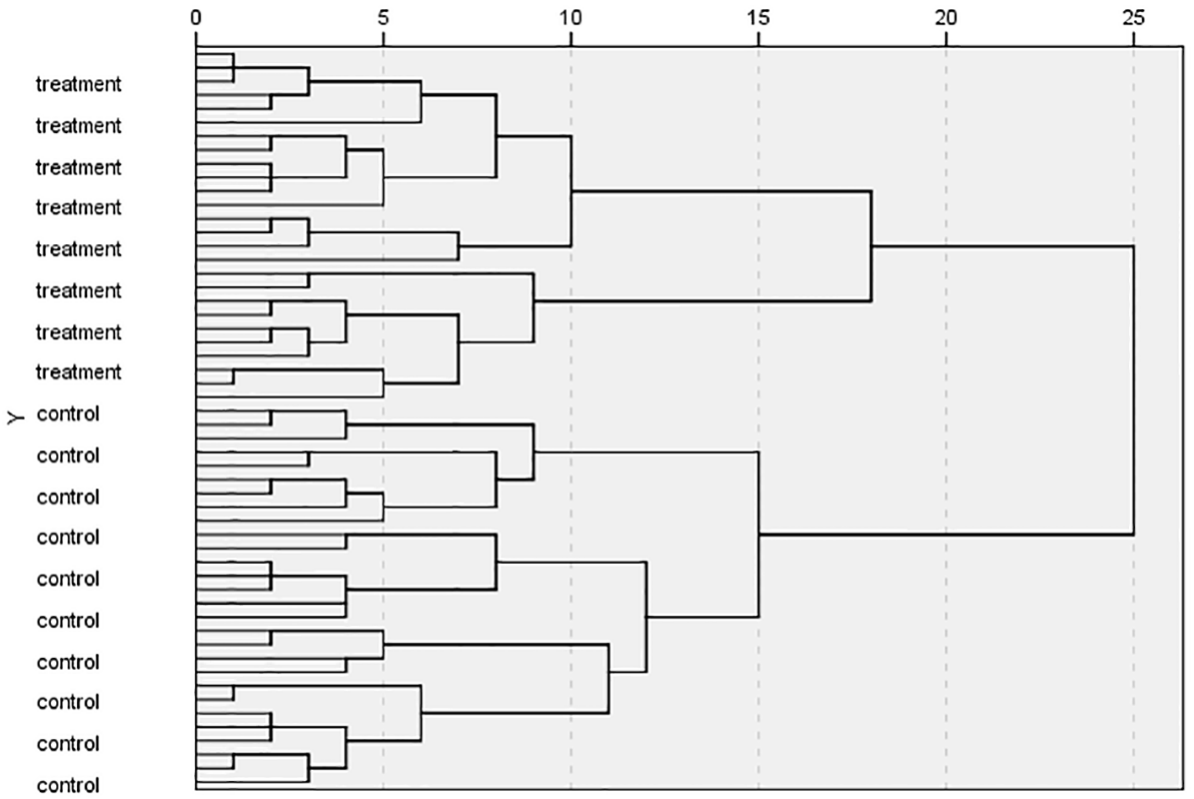
HCA results of eight genes.

To validate the PCA results, hierarchicalcluster analysis (HCA) was conducted for the same data. The results were shown in Fig 2, where "treatment" indicated test samples and "control" indicated control samples. All test samples were classified into a cluster and all control samples were classified into another, validating the PCA results.

The PCA and HCA results indicated that the group of genes selected by RNA-seq could be used to differentiate samples in the test and control groups and displayed an improvement on the results of nine genes selected by literatures. However, these genes in this study may not be used as potential biomarkers for monitoring drug treatment since genes are selected based on RNA-seq and biostatistical methods, further verification is required in other issues.

#### 3.1.4. qRT-PCR for the screened results by sequencing in combination with drug mechanisms

**Fig 3:**
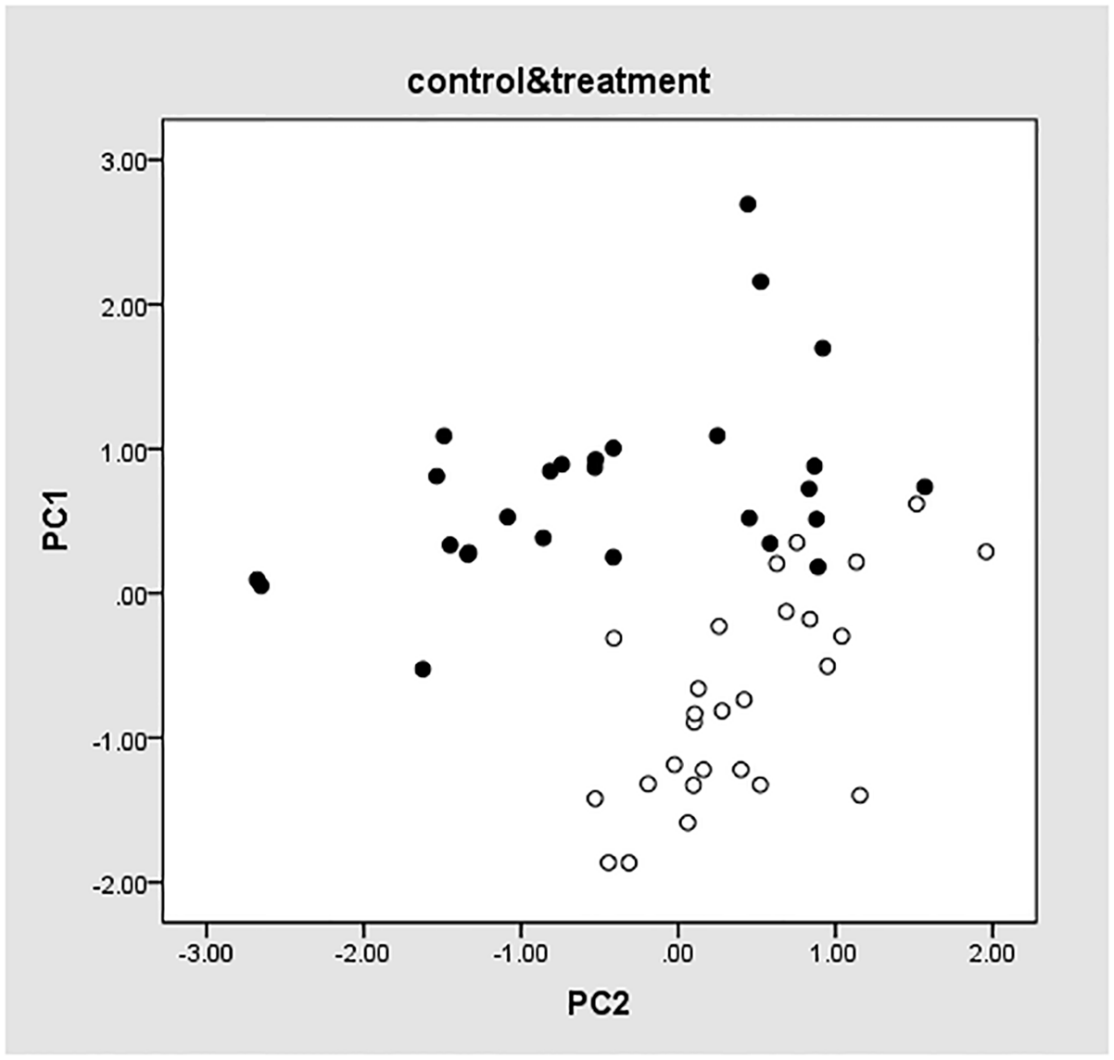
PCA values of 17 genes.

PCA was performed based on the eight genes selected by RNA-seq and nine genes selected from literatures. The results were shown in Fig 3, in which hollow points indicate experimental group samples, and solid points display control group samples. PC1 and PC2 are the obtained PCs. PC1 ranges from -0.51 to 2.68 for the control samples and -1.96 to 0.52 for the test samples. As shown in the figure, the two groups of samples are classified into separate clusters despite a few samples in the border areas. This demonstrated that the selected 17 genes could differentiate samples in the two groups, which slightly improved the test result using only nine genes selected from literatures.

**Fig 4:**
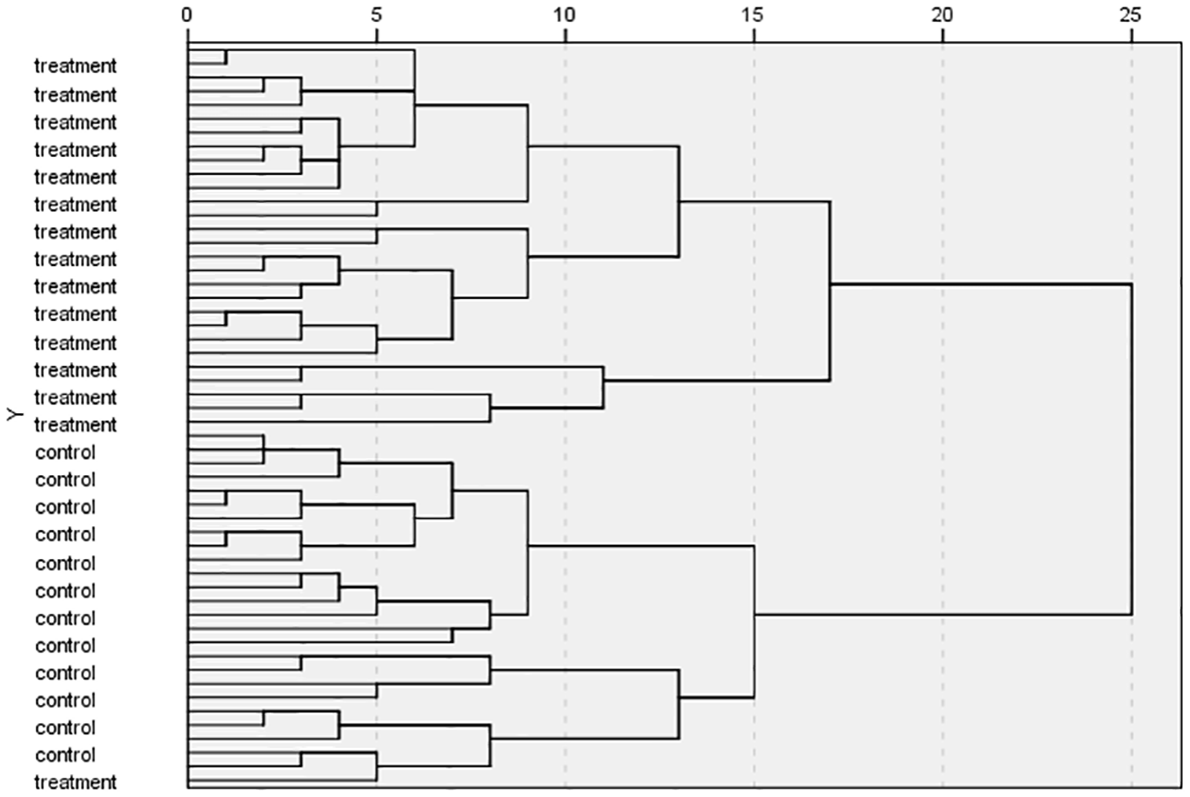
HCA results of 17 genes.

The HCA results were shown in Fig 4. As shown in Fig 4, all samples in the control group are in the first cluster; most samples in the treatment group are in the second cluster although a few ones are deviated from this cluster. Compared with the nine genes selected from literatures, the results using 17 genes witnessed an obvious improvement.

The DA results were shown in Table 3, in which 96.3% of the samples were correctly classified. The value was higher than nine genes, which had an accuracy of 96.3% and 92.6% for the initial classification procedure and subsequent cross-verification procedure, respectively.

**Table 3:** DA results of 17 genes.

Classification					
		Cluster	Prediction group member		Total
			Control	Test	
**Initial**	**Count**	**1**	**26**	**1**	**27**
		**2**	**1**	**26**	**27**
	**%**	**1**	**96.3**	**3.7**	**100.0**
		**2**	**3.7**	**96.3**	**100.0**
**Cross-verification**	**Count**	**1**	**26**	**1**	**27**
		**2**	**1**	**26**	**27**
	**%**	**1**	**96.3**	**3.7**	**100.0**
		**2**	**3.7**	**96.3**	**100.0**

In summary, the PCA results of 17 genes were slightly improved over those of 9 genes. Based on the same data, HCA and DA results using 17 genes indicated a significant improvement when compared with 9 genes. Therefore, the results of 17 genes selected by a combination of targeted and non-targeted methods as biomarkers to identify animals treated with ractopamine were more satisfactory. This study revealed that the method established in the earlier literature [20] could be inhanced by adding eight genes selected by RNA-seq, which has a high throughput potential.

### 3.2. Screening of more concise genes from 17 genes

Addition of eight genes selected by RNA-seq could optimize the earlier method, but more biomarkers mean heavier workload and consumption of more money and materials, which is a drawback to the application and implementation of this method. To further optimize the method and search for best-performance biomarkers, we used VIP to screen modulating genes that have critical influences on the model.

**Fig 5:**
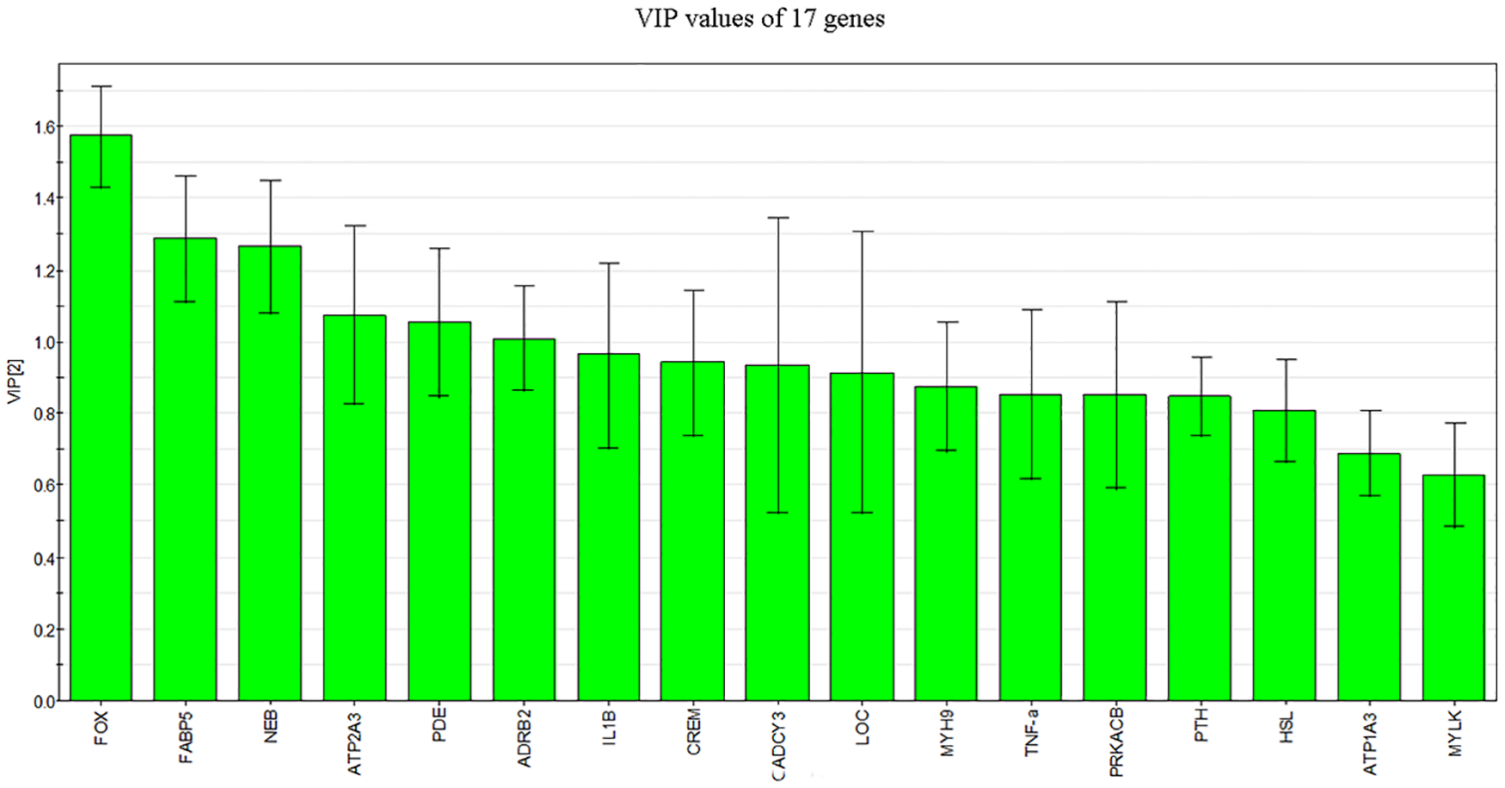
VIP illustration.

To screen key genes from the selected genes, SIMCA-P was used to conduct VIP. The VIP value greater than 1 is considered greatly influential on the model, and the impact of genes with VIP<1 on the model is negligible. As shown in Fig 5, six genes were selected, including FOX, FABP5, NEB, ATP2A3, PDE, and ADRB2, all of which have a VIP value of greater than 1.

**Fig 6:**
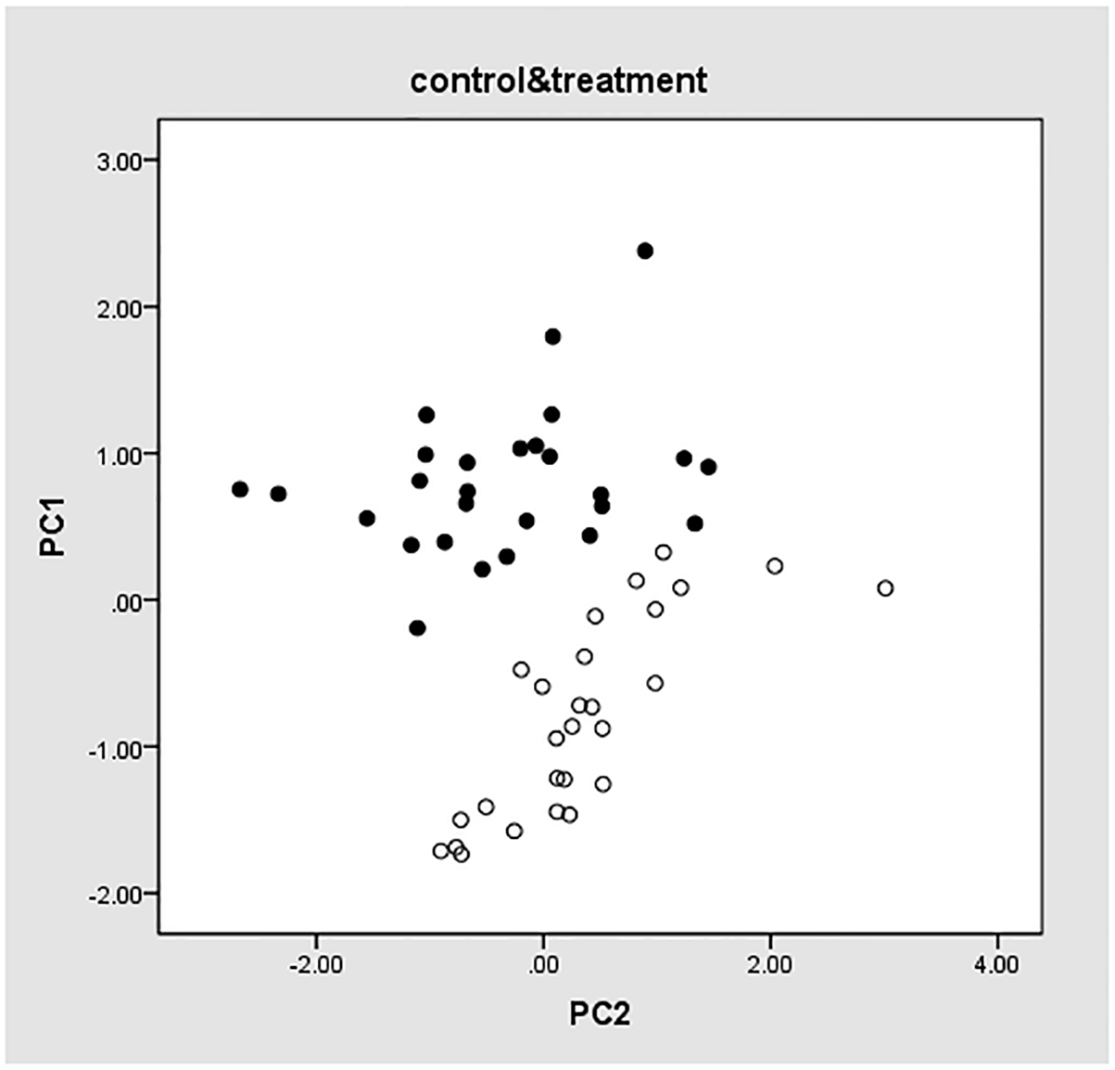
PCA values of six genes.

The PCA results for all the samples in the test and control groups using the preceding six genes were shown in Fig 6. PC1 and PC2 are the two PCs, and solid and hollow points represent the control and test group samples, respectively. As shown in the figure, PC1 ranged from -1.78 to 0.34 for the test group and -0.23 to 2.46 for the control group, which could be used to obviously differentiate the two groups, in concordant with the results in Fig 3. The PCA results indicated that the test results using the screened six genes were consistent with those based on the 17 genes, revealing that the six genes were the key factors and could be used to predict the model.

**Fig 7:**
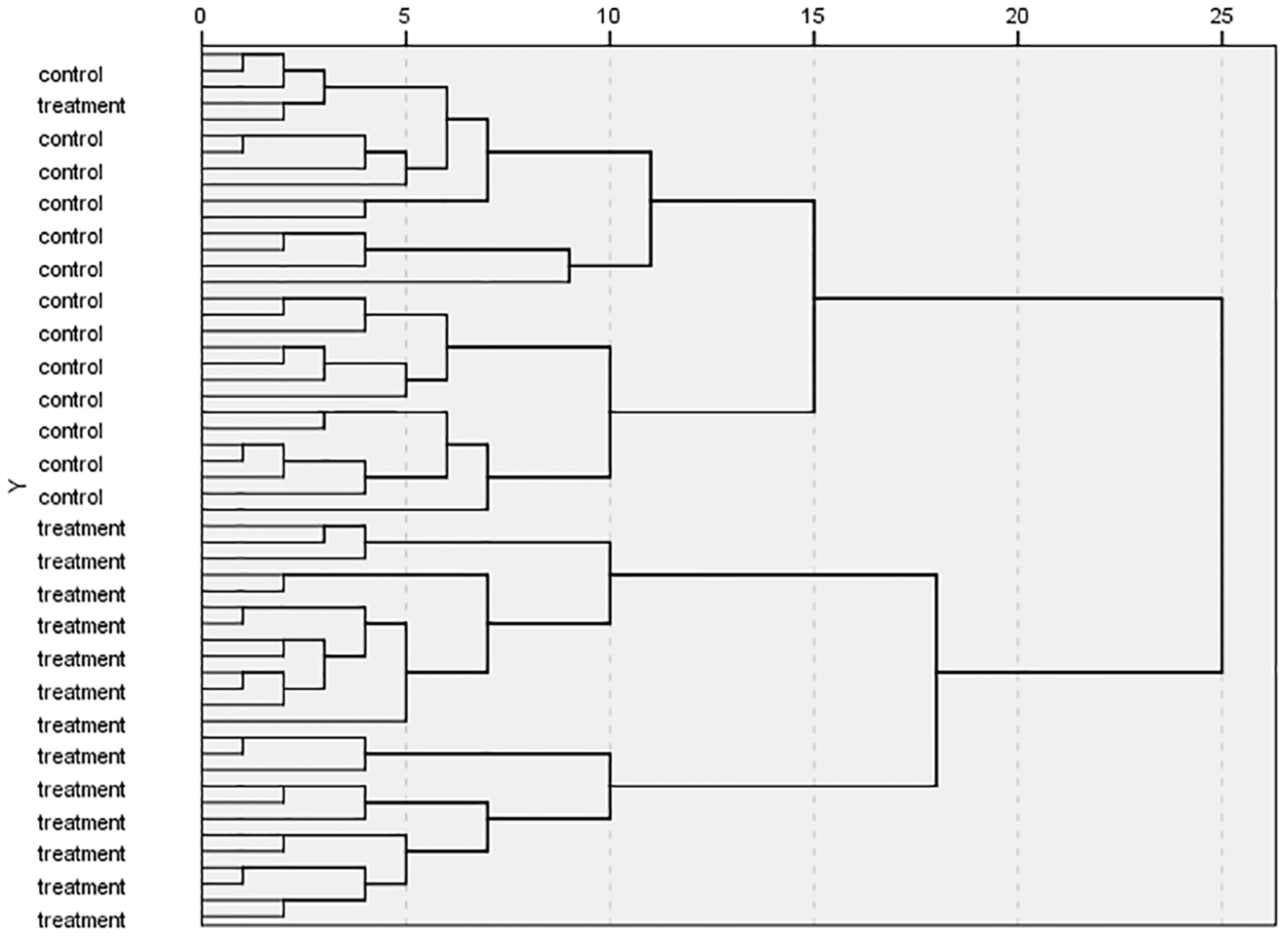
HCA results of six genes.

To validate the PCA results, HCA was carried out on the same data, and the results were shown in Fig 7. In this figure, "treatment" and "control" represent the samples in the test and control groups, respectively. According to the figure, control samples are classified into a cluster, and test group samples are classified into another cluster except one outlier included in the control cluster. This result was in agreement with that in Fig 4. This result also corresponded to the PCA results, displaying a great potential of the VIP method in screening key biomarkers.

DA was further carried out on the same data, and the results were shown in Table 4. By using the screened six genes, 96.3% of the samples were correctly classified during the initial classification procedure as well as the subsequent cross-verification procedure. This result completely agreed with Table 3.

**Table 4:** DA results of 6 genes.

Classification					
		Cluster	Prediction group member		Total
			Control	Test	
**Initial**	**Count**	**1**	**26**	**1**	**27**
		**2**	**1**	**26**	**27**
	**%**	**1**	**96.3**	**3.7**	**100.0**
		**2**	**3.7**	**96.3**	**100.0**
**Cross-verification**	**Count**	**1**	**26**	**1**	**27**
		**2**	**1**	**26**	**27**
	**%**	**1**	**96.3**	**3.7**	**100.0**
		**2**	**3.7**	**96.3**	**100.0**

DA results were given as the same as the data obtained by using 17 genes, presenting the criticality of these six genes. It should be noted that 100% accuracy in discriminating the samples could not be realized even though both targeted and non-targeted methods were used. Therefore, this group of screened genes should be further optimized to find out reliable and sensitive biomarkers to solve the regulation problem in drug misuse.

### 3.3. Verification of the six key genes

#### 3.3.1. RNA integrity

Agilent 2100 Bioanalyzer (Agilent, CA, US) was used to determine RNA integrity, and the results showed that the RIN values were between 7.5 and 8.5, indicating satisfaction of qRT-PCR requirements with the RNA integrity.

#### 3.3.2. Prediction of unknown samples based on the six key genes

DA was conducted on 25 samples, and the results were presented in Table 5. According to this table, 22 samples were classified into the control group with the prediction of non-drug-treated, and three samples were classified into the experimental group with opposite prediction. These possibly drug-treated samples were respectively labeled b-18, b-20, and b-24.

**Table 5:** DA results of 25 samples.

Classification					
		Cluster	Prediction group member		Total
			Control	Test	
**Initial**	**Count**	**1**	**26**	**1**	**27**
		**2**	**1**	**26**	**27**
		**Unclassified**	**22**	**3**	**25**
	**%**	**1**	**96.3**	**3.7**	**100.0**
		**2**	**3.7**	**96.3**	**100.0**
		**Unclassified**	**88.0**	**12.0**	**100.0**
**Cross-verification**	**Count**	**1**	**26**	**1**	**27**
		**2**	**1**	**26**	**27**
	**%**	**1**	**96.3**	**3.7**	**100.0**
		**2**	**3.7**	**96.3**	**100.0**

**Fig 8:**
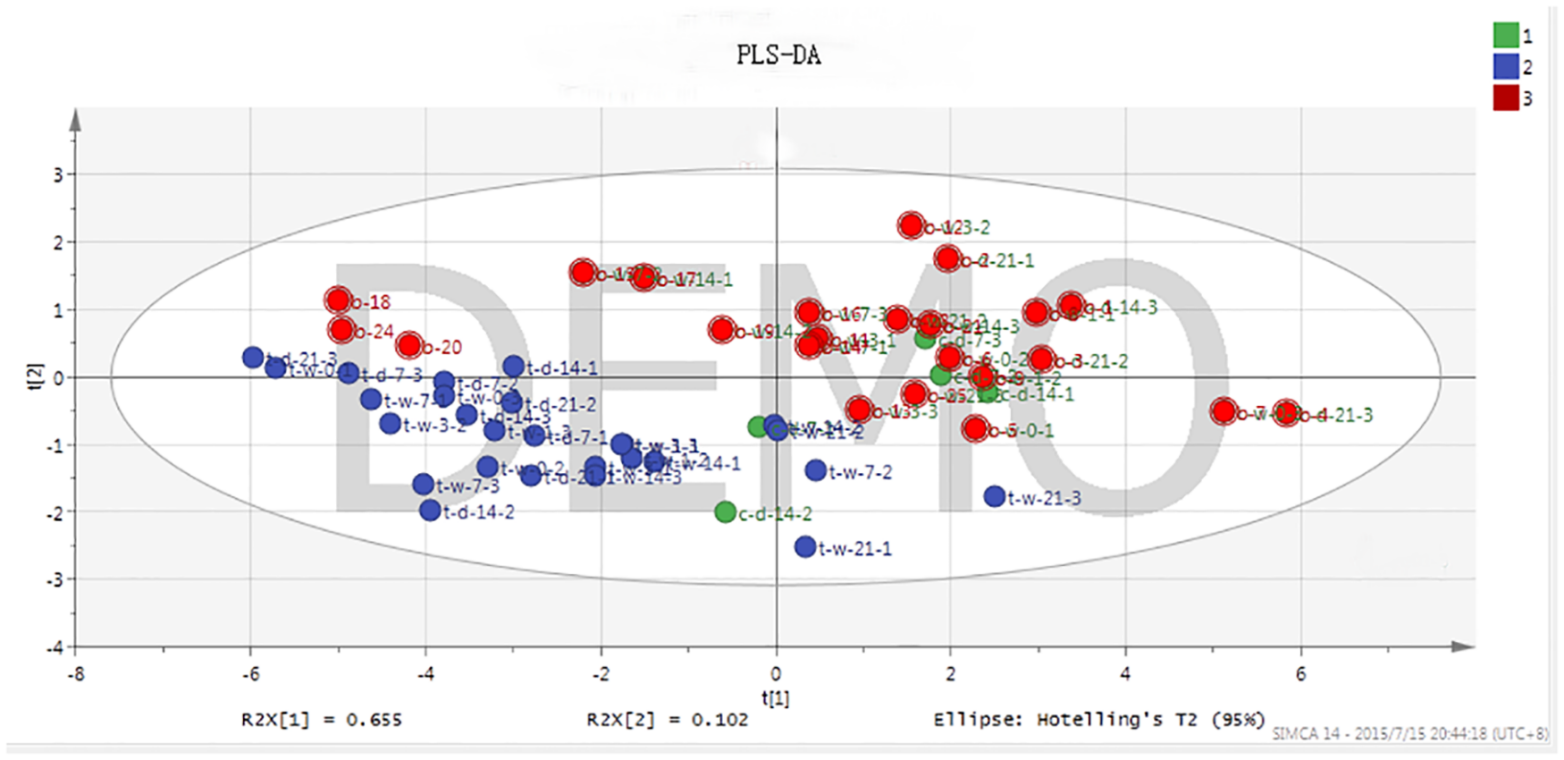
PLS-DA results of 25 samples.

To verify the accuracy of DA results, PLS-DA was conducted as a supervised analysis method to predict the 25 samples. The results were shown in Fig 8, in which three colors indicate different groups: color 1 represents the control group; color 2 is the experimental group; color 3 demonstrate the group containing undetermined samples. As shown in Fig 8, control and test groups are clearly divided, validating the accuracy of PCA and HCA results; most of the undetermined samples aggregate in the control group or even overlap with the control samples, indicating they are non-drug-treated samples which possess same information as control samples; samples b-18, b-20, and b-24 aggregate in the experimental group, indicating that they are drug treated. PLS-DA and DA had concordant results, demonstrating that samples b-18, b-20 and b-24 were determined as drug-treated samples. However, the results required further verification.

#### 3.3.3. Instrument-assisted verification of the prediction results using the six key genes

To identify the use of β_2_-agonists with biomarker of the six genes, instruments were used to measure β_2_-agonist drug residues in the three suspected samples determined by DA and PLS-DA. For detailed operations, see [Detection of β-receptor agonist residues in animal-source food by LC-MS/MS (Notice #1025-18-2008, Ministry of Agriculture, China)]. With the addition concentration of 0.5 μg/kg to 2 μg/kg, the recovery rates were between 70% and 120%. Three positive samples were measured, and mapenterol, a type of β_2_-agonist, was found in the sample labeled b-24, with the concentration of 0.772 μg/kg (Y = 1309.9X + 480.18, R^2^ = 0.99868).

In this part, unsupervised method DA and supervised method PLS-DA were used to predict drug treatment of 25 undetermined samples. Both methods determined the same three positive samples, which were labeled b-18, b-20 and b-24. The β_2_-agonist residue of was detected only in the sample labeled b-24, which might be caused by instrument sensitivity deficiency. Maybe the drug was completely decomposed but it still had some effects. There is another assumption that b-18 and b-20 samples do not contain β_2_-agonists but contain drugs that have similar physiological effects, which could present false-positive results.

Mapenterol was detected from a suspected sample. β_2_-agonists are structurally divided into aniline and phenol types. In this study, ractopamine is a phenol type, while mapenterol is an aniline type, indicating that this group of genes can be used to monitor β_2_-agonist drugs.

The instrument-aided verification result indicated that prediction of unknown drug-treated samples using the six genes screened by literatures, RNA-seq, and VIP process was correct. This group of genes can be used as biomarkers to monitor the abuse of β_2_-agonists, which is practicable and effective. However, to determine more stable and sensitive biomarkers at the transcriptional level, this group of genes needs further optimization. For example, animal samples treated with a longer period of drug withdrawal are needed, and samples of different tissues, species, and genders are required for the study. These will facilitate monitoring of β_2_-agonists misuse in animal husbandry.

In this study, six key genes were selected to monitor β_2_-receptor agonist misuse in animal husbandry as biomarkers. PCA, HCA, and DA results indicated that eight genes selected by high-throughput RNA-seq technique plus nine genes selected in earlier literatures collaboratively achieved better performance, demonstrating the accuracy of the selected genes by RNA-seq. The results using six key genes selected from the 17 genes were consistent with the results of 17 genes, revealing that the six genes were the critical ones. The instrumental verification showed the correctness of the results predicted on the undetermined samples by the six genes, validating the precision and reliability of the six key genes. With both RNA-seq and earlier literatures, detection accuracy can be improved by finding key genes. Then effectiveness of the genes was verified, which imposed a great importance on effective monitoring of β_2_-agonist drugs abuse in animal husbandry.

## 4. Discussion

Transcriptomics is extensively studied for monitoring β_2_-agonists misuse in current animal husbandry, making it possible to determine the physiological functional impacts of the drugs at the molecular level [24]. Generally, gene expression quantity changes are measured by qRT-PCR, a highly sensitive method with a wide quantification range. Compared with chip techniques, this method is highly repetitive with a low cost, in addition to easy operations and analysis of multiple biological species of samples in one cycle. Therefore, qRT-PCR is widely used for the study of biomarkers in transcriptomics [25]. Compared with qRT-PCR, microarray technique allow analysis of a set of complete genes in an array [26,27]. However, the latest high-throughput RNA-seq method is more sensitive than the microarray approach, with a higher probability of finding more biomarkers.

In a previous research, a targeted -omics method, that is, screening according to the literatures, was used to screen target genes. After verification by qRT-PCR, statistical results showed that selected genes could be used as biomarkers to differentiate ractopamine-treated and untreated animals. To further optimize the method, this study used a non-targeted -omics technique, RNA-seq, to screen possible biomarkers in combination with earlier study by exploiting great potential of RNA-seq in searching for transcriptional biomarkers. This aim to find out optimal biomarkers and apply them in actual monitoring. As a result, the screened 17 genes brought in an improvement compared with previous nine genes, demonstrating the superiority of RNA-seq. The combination of targeted and non-targeted -omics techniques is a better method for searching for optimal biomarkers.

Biostatistical methods such as PCA, HCA and DA have a strong potential and are extremely important in screening potential biomarkers to differentiate experimental samples from control samples. Now, these methods have been widely used in screening biomarkers at various levels, and biomarkers selected based on VIP have also been extensively used in -omics studies [28–31].

Among the six genes, ATP2A3, PDE and ADRB2 are key factors for the β_2_-agonists signal pathway, and FOX, FABP5 and NEB are also associated with the physiological functions of β_2_-agonists, indicating that the changes in gene expression levels are specific to the treatment of β_2_-agonists. In the earlier research, candidate genes were selected based on the mechanisms of β_2_-agonists, but the animals in the test group were not treated with other β_2_-agonist drugs, and therefore the selected genes maynot be used as biomarkers to monitor β_2_-agonists except ractopamine. In this study, another type of β_2_-agonist mapenterol was detected in a potentially drug-treated sample after prediction of the six genes, demonstrating this group of genes could realize the monitoring of all β_2_-agonist drugs.

To determine more stable and sensitive biomarkers at the transcriptional level in the long run, this group of genes needs to be optimized. For example, animal samples treated with a longer period of drug withdrawal are needed, and samples of different tissues, species, and genders are needed. These will facilitate monitoring of β_2_-agonists misuse in animal husbandry.

## 5. Conclusion

In this study, RNA-seq was used as a non-targeted-omics technique to optimize the group of genes selected through a targeted -omics method. There were 17 genes with more information combining a targeted -omics method and a non-targeted -omics technique. PCA, HCA and DA results indicated that eight genes selected by high-throughput RNA-seq plus nine genes obtained from literatures collaboratively achieved better performance, demonstrating the accuracy of the RNA-seq-selected genes. Since 17 genes possessed better results than nine genes, more genes mean more work in actual monitoring. To further optimize the method, six genes carrying key information in the 17 genes with VIP>1 were extracted upon a certain algorithm (VIP value). The test results using the six key genes were consistent with the results of 17 genes, revealing that the six genes were the critical genes. The six key factors were then employed to predict whether drugs were used in 25 unknown samples through DA and PLS-DA. LC-MS/MS applied to verify the positive samples showed that the results predicted on the positive samples by the six genes were accurate and reliable. The selected six key genes possess equal accuracy and information with 17 genes mean less amount of operation in actual monitoring, imposing a great importance in effective and efficient monitoring of β_2_-agonist drugs abuse in animal husbandry.

## Supporting information

S1 Table β_2_-agonist drugs and their MS parameters. (DOC)
