# Supplementary material for: Identification of transcriptional biomarkers by RNA-sequencing for improved detection of β2-agonists abuse in goat skeletal muscle
Source: PLoS One. 2017 Jul 26;12(7):e0181695. doi: 10.1371/journal.pone.0181695 (PMC5528896; doi:10.1371/journal.pone.0181695)
Supplement: S1 Table — (DOC) [file pone.0181695.s001.doc]

**Table S1.**

| **Analyte** | **Parent ion**  **Q1 (m/z)** | **Child ion**  **Q3 (m/z)** | **DP/V** | **CE/V** | **Retention time (min)** |
| --- | --- | --- | --- | --- | --- |
| **terbutaline** | **226.15** | **151.74^a^;124.67** | **25** | **15;22** | **1.94** |
| **cimaterol** | **220.18** | **201.95^a^;129.77** | **20** | **10;16** | **1.98** |
| **salbutamol** | **247.17** | **147.70^a^;221.97** | **22** | **18;10** | **2.08** |
| **fenoterol** | **304.15** | **106.59^a^;134.61** | **35** | **18;30** | **3.83** |
| **clorprenaline** | **214.13** | **153.75^a^;195.97** | **25** | **18;12** | **4.81** |
| **ractopamine** | **302.33** | **163.87^a^;106.77** | **25** | **15;28** | **4.96** |
| **clenbuterol** | **277.11** | **202.78^a^;258.94** | **25** | **15;10** | **4.89** |
| **tulobuterol** | **228.22** | **153.90^a^;171.88** | **25** | **15;12** | **5.39** |
| **penbutolol** | **292.36** | **236.22^a^;201.00** | **30** | **15;20** | **8.76** |
| **cimbuterol** | **234.16** | **216.15^a^;160.08** | **23** | **17;15** | **3.63** |
| **zilpaterol** | **262.15** | **202.09^a^;185.07** | **25** | **25;20** | **6.73** |
| **clenproperol** | **263.07** | **203.01^a^;132.06** | **20** | **20;20** | **2.30** |
| **mabuterol** | **311.11** | **217.03^a^;237.03** | **20** | **10;20** | **2.92** |
| **labetalol** | **329.15** | **311.17^a^;162.05** | **20** | **20;35** | **5.38** |
| **bambuterol** | **368.22** | **312.15^a^;249.08** | **30** | **30;15** | **5.01** |
| **salmeterol** | **416.28** | **232.16^a^;248.16** | **20** | **15;25** | **5.45** |
| **mapenterol** | **325.12** | **217.03^a^;237.04** | **25** | **20;15** | **5.71** |
| **ritodrine** | **388.15** | **150.09^a^;121.06** | **25** | **18;20** | **3.92** |
| **formoterol** | **345.18** | **121.06^a^;149.09** | **25** | **20;25** | **4.84** |

**β_2_-agonist drugs and their MS parameters**

**^a^ quantification ion**
